# Testing a physical education-delivered autonomy supportive intervention to promote leisure-time physical activity in lower secondary school students: the PETALS trial

**DOI:** 10.1186/s12889-020-09518-3

**Published:** 2020-09-22

**Authors:** Jekaterina Schneider, Juho Polet, Mary Hassandra, Taru Lintunen, Arto Laukkanen, Nelli Hankonen, Mirja Hirvensalo, Tuija H. Tammelin, Timo Törmäkangas, Martin S. Hagger

**Affiliations:** 1 Faculty of Sport and Health Sciences, University of Jyväskylä, Keskussairaalantie 4, 40600 Jyväskylä, Finland; 2 Department of Physical Education and Sport Science, University of Thessaly, Thessaly, Greece; 3 Faculty of Social Sciences, University of Helsinki, Helsinki, Finland; 4 LIKES Research Centre for Physical Activity and Health, Jyväskylä, Finland; 5 Psychological Sciences, University of California, Merced, USA

**Keywords:** Autonomous motivation, Autonomy support, Physical activity, Physical education, Trans-contextual model

## Abstract

**Background:**

Inadequate physical activity in young people is associated with several physical and mental health concerns. Physical education (PE) is a potentially viable existing network for promoting physical activity in this population. However, little research has been conducted on whether PE teachers can influence students’ engagement in leisure-time physical activity. The present study therefore examined the efficacy of an intervention aimed at increasing PE teachers’ autonomy support on students’ leisure-time physical activity (the PETALS trial). The intervention was guided by the trans-contextual model (TCM) explaining the processes by which PE teachers’ provision of autonomy support during PE promotes students’ motivation and engagement in physical activity in their leisure time.

**Methods:**

The study adopted a cluster-randomized, waitlist control intervention design with randomization by school. Participants were PE teachers (*N* = 29, 44.83%female; *M* age = 42.83, *SD* = 9.53 yrs) and their lower secondary school students (*N* = 502, 43.82%female; *M* age = 14.52, *SD* = 0.71 yrs). We measured TCM constructs, including perceived autonomy support, autonomous motivation in PE and leisure time, beliefs and intentions towards leisure-time physical activity, and physical activity behavior at baseline, post-intervention, and at one-, three-, and six-months. Study hypotheses were tested through a series of ANOVAs and structural equation models using post-intervention and one-month follow-up data.

**Results:**

We found no changes in TCM constructs or physical activity behavior in either group at post-intervention or at 1 month. Path analyses supported two propositions of the TCM as change variables: perceived autonomy support had a significant effect on autonomous motivation in PE and autonomous motivation in PE had a significant effect on autonomous motivation in leisure time. Although we found a direct effect of autonomous motivation in leisure time on physical activity, we did not find support for the third premise of the TCM that autonomous motivation in leisure time indirectly affects physical activity through beliefs and intentions.

**Conclusions:**

Current findings did not support the efficacy of the PETALS intervention at changing physical activity behavior and TCM constructs. More research is required to determine whether the TCM predictive validity is supported when other model variables are manipulated through experimental and intervention studies.

**Trial registration:**

ISRCTN, ISRCTN39374060. Registered 19 July 2018. Prospectively registered.

## Introduction

Insufficient physical activity in young people is associated with several physical and mental health problems, such as increased rates of juvenile obesity, cardiovascular disease risk factors, and prevalence of depressive symptoms and psychological distress, and reduced psychological well-being and overall quality of life [1–3]. Conversely, regular physical activity is associated with reduced disease risk and adaptive mental health outcomes [4, 5]. Evidence also suggests that physical activity during adolescence may be an antecedent of an active lifestyle in adulthood [6]. However, international [7] and national [8] studies have shown that the majority of young people do not achieve the recommended physical activity levels to confer health benefits [9]. Furthermore, participation in physical activity tends to decrease throughout childhood, with particularly steep declines observed during adolescence [10, 11]. National trends in Finland suggest that 45% of 11-year old students meet guideline levels for physical activity, while only 19% of 15-year olds achieve these levels [8].

Given the importance of promoting physical activity among young people, researchers have sought to identify effective strategies and contexts to enhance physical activity in this population [12]. Physical education (PE) has been identified as a potentially viable existing network through which physical activity interventions can be delivered to a broad, diverse, and captive audience of young people [13, 14]. However, relatively little research has been conducted on the extent to which in-school PE can influence students to engage in physical activity out-of-school. PE teachers are important ‘frontline practitioners’ that can provide support for, and foster motivation toward, physical activity participation among school students during PE classes, but can also be influential in promoting physical activity outside of school. The promotion of leisure-time physical activity is important because physical activities performed during PE are unlikely to be sufficient for students to meet national physical activity guidelines. As such, research has been increasingly focused on examining factors related to young people’s motivation toward physical activity both within [15] and outside of school [16]. Identifying the determinants of engagement in physical activity in school may assist in informing potential intervention strategies delivered in PE that promote leisure-time physical activity in children and adolescents [17–19].

A prominent means of identifying the determinants of in-school and out-of-school physical activity is through the application of social psychological and motivational theories [20]. While much of this work has focused on identifying the determinants of physical activity in PE (e.g., [15, 21]), several studies have also examined how school students’ motivation toward physical activity fostered in a PE context relates to physical activity participation in a leisure-time context [22]. A theoretical model that was developed explicitly to identify the physical activity determinants on these contexts is the trans-contextual model (TCM) [22]. The model has been applied consistently to identify the motivational and belief-based determinants of physical activity participation among schoolchildren in PE and leisure-time contexts. However, there is a relative dearth of research that has used the model as a basis for interventions administered in a PE context aimed at changing school students’ physical activity performed in their leisure time. This study aimed to fill this evidence gap by testing the efficacy of a school PE-delivered intervention based on the TCM in promoting leisure-time physical activity among Finnish lower secondary school students.

### The trans-contextual model

The TCM is a multi-theory model comprising constructs and hypotheses from three social psychological theories to identify the determinants of school students’ motivation toward, and engagement in, leisure-time physical activity [22]. The TCM draws its propositions from self-determination theory [23], the theory of planned behavior [24], and the hierarchical model of intrinsic and extrinsic motivation [25].

The concept of autonomous motivation from self-determination theory is central to the TCM. Autonomous motivation is a form of motivation reflecting self-endorsed reasons for acting, such that behaviors are experienced as chosen and originating from the self. Autonomously motivated individuals engage in activities out of personal interest, choice, and volition. Importantly, autonomous motivation is related to behavioral persistence across populations, behaviors, and contexts regardless of external reinforcements and contingencies [26]. In the TCM, promoting students’ autonomous motivation in PE is considered important to promote their persistence on activities in that context. According to the theory, teachers are integral to fostering autonomous motivation in PE through the autonomy supportive behaviors they display. Research has indicated that students whose PE teachers display autonomy supportive behaviors show, on average, more autonomous motivation in class, and greater persistence on class tasks and activities [27, 28]. Furthermore, students that *perceive* their teachers as autonomy supportive are also more likely to report autonomous motivation in class [29–31]. These predictions form the first premise of the TCM: students who perceive that their PE teacher supports their autonomy in class will be autonomously motivated towards physical activities in the PE context.

Another important premise of the TCM is the trans-contextual relationship between students’ autonomous motivation toward physical activities across PE and leisure-time contexts. This premise is based on Vallerand and Ratelle’s hierarchical model of intrinsic and extrinsic motivation [25] that proposes a ‘transfer’ of motivation across different contexts, such that individuals who experience activities as autonomously motivating in one context are also more likely to seek out similar autonomously motivating activities in other contexts. Basic psychological needs of autonomy, competence, and relatedness from self-determination theory [32] are proposed to be the driving force behind this trans-contextual relationship. Accordingly, this forms the second premise of the TCM: students who experience physical activity as autonomously motivating in school PE will also be autonomously motivated towards physical activity in their leisure time.

Finally, the TCM draws from the theory of planned behavior [24] in identifying the proximal belief-based determinants of future behavior participation. Specifically, the model proposes that intention is the most immediate determinant of behavior [33, 34], and intentions are a function of three sets of belief-based constructs: attitudes, which reflect individuals’ positive and negative evaluations of a target behavior; subjective norms, which reflect individuals’ beliefs that significant others want them to participate in the behavior; and perceived behavioral control, which reflects individuals’ personal evaluations of their capacity to perform the behavior. The utility of including the belief-based determinants from the theory of planned behavior in the TCM is that they provide a link between the motives from self-determination theory and participation in leisure-time physical activity. Consistent with original predictions of self-determination theory, individuals with autonomous motives toward need satisfying behaviors will seek out those behaviors in the future. Research has shown that when individuals experience a given behavior as autonomously motivating, they will strategically align their beliefs and intentions regarding that future behavior with their motives [35, 36]. A meta-analysis [37] and a recent panel study [38] have supported these predictions. This forms the third premise of the TCM: effects of autonomous motivation toward leisure-time physical activity on leisure-time physical activity engagement will be mediated by intentions and the belief-based constructs from the theory of planned behavior.

### Interventions based on the trans-contextual model

The TCM provides a theoretical framework for developing interventions in school PE, to promote motivation towards, and engagement in, leisure-time physical activity. The three premises of the TCM have received substantial empirical support in multiple studies and settings [37, 39–41]. The proposed model effects are supported by research demonstrating the premises of the model, including the trans-contextual effects and the mechanisms involved [37]. The model therefore signals potentially modifiable determinants that can be targeted for change by the content of behavior change interventions. A key target suggested in the model is students’ perceived autonomy support from their PE teacher. Increasing perceived autonomy support is likely to enhance students’ motivation toward, and engagement in, physical activity in their leisure time through the mediation of the TCM constructs [42]. A recent study has provided additional support for this, demonstrating that change in model constructs over time results in change in motivation and physical activity engagement [43].

Interventions based on the TCM require changes in the behavior of PE teachers in order to affect changes in students’ motivation and behavior toward physical activities performed in PE and in leisure time. Specifically, the interventions require training of teachers in adopting autonomy supportive behaviors, and for them to implement these behaviors in their regular lessons over a period of time. Numerous autonomy supportive training programs exist, foremost among which are the autonomy supportive intervention programs developed by Cheon and Reeve [44]. These involve instruction and practice over a period of weeks of autonomy supportive behaviors, such as providing rationale, adopting a questioning approach, assisting rather than telling students when solving problems, active listening to students, positive feedback, and avoiding controlling language. These techniques have been formalized in taxonomies of behavior change techniques from self-determination theory and other motivational perspectives [45, 46]. After teachers receive the autonomy supportive training, the implementation period in which teachers apply the autonomy supportive behaviors they learned in the program is proposed to foster autonomous motivation in students toward the activities in class and outside of school, consistent with the premises of the TCM. Research supports the application of autonomy supportive intervention programs in promoting autonomous motivation and behavior change in academic contexts [28].

Although there are relatively few studies to date, there is growing evidence for the use of the TCM as a basis for guiding physical activity interventions. Studies applying the TCM have demonstrated that promoting autonomy support in PE through PE teacher autonomy support training leads to observed changes in autonomous motivation, the theory of planned behavior constructs, and actual leisure-time physical activity engagement outside of school [47–49]. Importantly, the research provides preliminary evidence that the intervention increases physical activity engagement mediated by the proposed constructs of the TCM [47]. These studies notwithstanding, there is a need for further intervention and experimental research testing the efficacy of the model within PE-based interventions.

### The present study

**Fig. 1:**
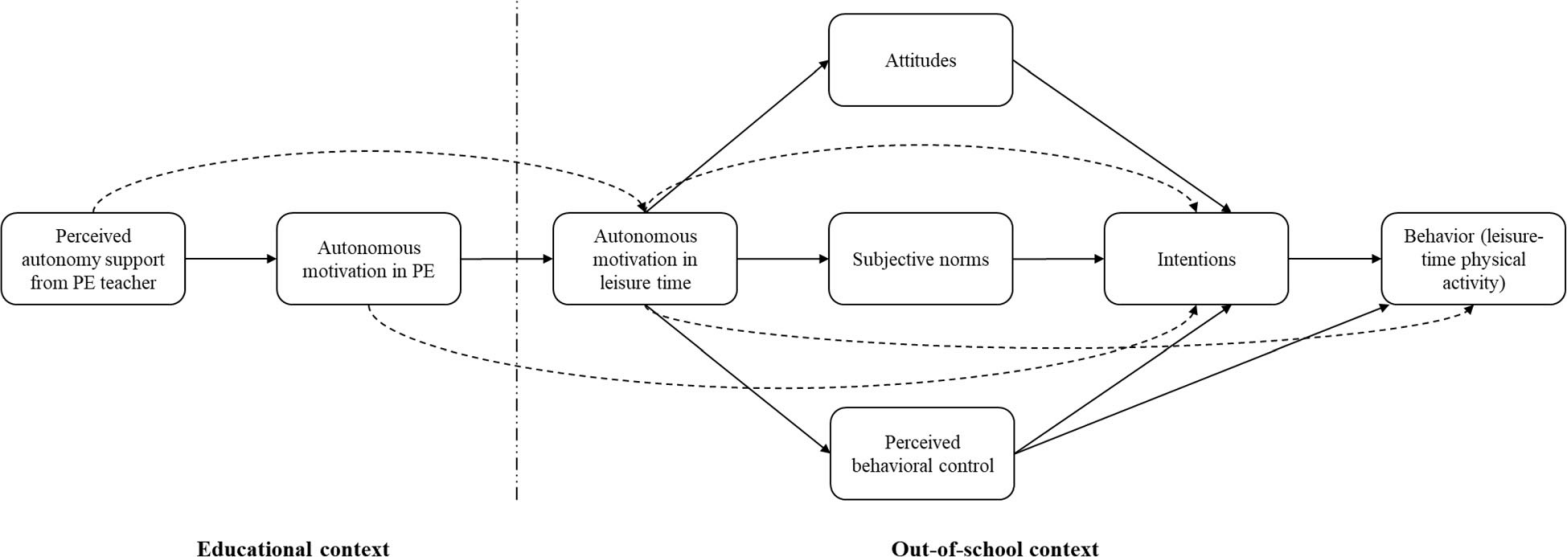
Proposed Relations Among Constructs From the Trans-Contextual Model. Note. Broken lines between constructs indicate direct effects proposed to be non-significant or unsubstantial relative to the indirect effects. Changes in students’ perceived autonomy support from teachers in PE are proposed to be positively related to changes in autonomous motivation toward PE; changes in autonomous motivation in PE are proposed to be positively related to changes in autonomous motivation for leisure-time physical activity outside of school; changes in autonomous motivation for leisure-time physical activity are proposed to be positively related to changes in intentions toward, and actual participation in, subsequent leisure-time physical activity through changes in the immediate antecedents of intentions (i.e. attitudes, subjective norms, and perceived behavioral control); PE = Physical education

The present study examined the efficacy of a teacher-delivered autonomy supportive intervention based on the TCM in promoting leisure-time physical activity engagement in lower secondary school students. The intervention comprised PE teachers’ autonomy-support training to promote leisure-time physical activity in schoolchildren, known as the PETALS trial. Effects of the intervention were evaluated through changes in subsequent follow-up measures of physical activity levels among students in the teachers’ classes and TCM variables relative to pre-trial baseline measures. We hypothesized that students in schools whose teachers received the autonomy supportive intervention would exhibit greater engagement in physical activities in their leisure time relative to students in schools whose teachers did not receive the intervention. We also predicted that students in the intervention group would report higher levels on TCM constructs (perceived autonomy support from PE teachers, autonomous motivation in PE and leisure-time contexts, attitudes, subjective norms, perceived behavioral control, and intentions) relative to students in the control group (see Fig. 1). The trial protocol was prospectively registered (registration no. ISRCTN39374060) and the study protocol was published previously [50].

## Method

### Study design and procedure

This study adopted a cluster-randomized, waitlist control intervention design with randomization by school. Secondary school teachers from 11 schools and their selected students in the city of Jyväskylä were invited to participate in the study. The total potential eligible pool of students was 587. At baseline, all consenting teachers and students completed a questionnaire containing demographic, psychological, and behavioral measures. Baseline data collection was followed by the teacher-training and implementation phases of the trial. The teacher-training phase comprised a two-week, two times six-hour (altogether 12 h) training program in which the teachers received the autonomy support training program developed for the present study from professional PE teacher educators. Training was followed by a one-month implementation phase during which teachers were instructed to apply autonomy supportive techniques in their regular PE classes. Following the implementation phase, post-intervention data were collected comprising identical measures as at baseline, with the exception of demographic information. Follow-up data collection occasions were scheduled for one, three, and 6 months post-intervention. The waitlist control group received the autonomy support training program immediately before the three-month follow-up data collection occasion.^1^ Post-intervention and one-month follow-up assessments were conducted for teachers and students in the waitlist group, using identical measures as those administered in the intervention group. A depiction of the study timeline including key milestones and data collection occasions is presented in Fig. S1, Appendix A (supplementary materials).

### Participants

Participants were Finnish PE teachers (*N* = 29, 44.83% female; *M* age = 42.83, *SD* = 9.53 years) and their lower secondary school students (*N* = 502, 43.82% female; *M* age = 14.52, *SD* = 0.71 years), who were recruited through established links with schools across Jyväskylä and support from the Education Department of the city. Based on an a priori statistical power analysis [50], we aimed to recruit 476 student participants at baseline (*n* = 238 participants in each group) to detect effect sizes observed in previous TCM research. All available lower secondary level school PE teachers in the city participated in the teacher-training phase of the study, irrespective of their participation in the study, as the city’s Education Department had accepted the teacher training to be part of PE teachers’ regular in-service training. Qualified full-time PE teachers teaching regular PE lessons in lower secondary schools (*N* = 30) were eligible to participate in the study with one of their PE classes assigned to take part. Only one eligible teacher declined to participate. Students in grades 7 to 9 (typical ages 13 to 15 years) in lower secondary schools were eligible to participate in the study. Students with existing physical or mental health conditions that could prevent participation in PE lessons, regular physical activity, or completing surveys were excluded from the study. Informed consent procedures are described in Polet et al. [50].

### Intervention

#### Autonomy support intervention group

Teachers in schools allocated to the intervention condition received the 12-h interactive autonomy support teacher-training program developed specifically for this study [50], but informed by previous autonomy supportive intervention programs [28, 51]. The program aimed to familiarize PE teachers with techniques and strategies intended to promote students’ autonomous motivation toward out-of-school physical activities. The program focused on six sets of autonomy-supportive strategies and techniques: taking students’ perspective, using non-controlling and informational language, providing rationale, displaying patience, providing choices, and accepting negative emotions and feelings (see Table S1, Appendix B, supplementary materials), which were adapted from strategies identified in previous autonomy support training programs [51–53]. The training was delivered by two trained teacher trainers with extensive experience of PE teacher education. Full details of the schedule and content of the autonomy support training program with accompanying training materials are available online: https://osf.io/s4b2g/.

#### Waitlist control group

Participating teachers allocated to the waitlist control group received an alternative training program comprising 4 h of education on how to apply a monitoring system for physical functional capacity for children with special needs (MOVE) [54]. Two educators experienced in PE teacher training delivered the control intervention in a one-day workshop.

### Measures

Self-report measures were translated from English to Finnish using a back-translation process by two bilingual researchers [55]. Full details about each measure can be found in the protocol article [50]. A summary of study measures, data collection occasions, and methods is provided in Table S2, Appendix C (supplementary materials).

#### Outcome variables

The primary outcome variable was students’ leisure-time physical activity engagement, which was measured at baseline, immediately post-intervention, and at one-month post-intervention follow-up,^2^ using the short form of the International Physical Activity Questionnaire (IPAQ) [56], which was modified to make explicit reference to out-of-school physical activity. In addition, a subsample of participants (*n* = 122) covering grades 7 to 9 also had their physical activity measured using accelerometry (Hookie AM 20), to provide concurrent validity and comparison data for the IPAQ.

#### Measures of trans-contextual model constructs

All students completed a battery of self-report measures of psychological variables based on the TCM. These included: perceived autonomy support from PE teachers, measured using the Perceived Autonomy Support Scale for Exercise Settings [57]; autonomous and controlled forms of motivation in PE and in leisure time, measured using a modified version of the Perceived Locus of Causality Questionnaire [58] and the amotivation subscale from the Sport Motivation Scale [59]; and attitudes, subjective norms, perceived behavioral control, and intentions for physical activity, measured using scales developed according to recommended guidelines [60].

#### Teachers’ measures

Teachers’ self-reported provision of autonomy support to students in PE lessons was measured using an adapted version of the Perceived Autonomy Support Scale for Exercise Settings [57]. We also developed an additional item for the autonomy support scale to assess teachers’ self-reported provision of autonomy support for students’ engagement in leisure-time physical activity and provision of a rationale for students’ participation in PE. Similarly, teachers’ use of controlling behaviors in PE lessons was measured using an adapted three-item version of the Teacher Social Context Questionnaire [61].

#### Demographic variables

Participating PE teachers self-reported the following demographic details: age, gender, education, years of teaching experience, and number of students in their PE class. Students self-reported their age, grade, gender, and school. We also collected the following demographic details from participating parents: gender, nationality of child, ethnicity of child, and highest level of education.

### Data analytic strategy

Study hypotheses were tested through a series of structural equation models estimated by the M*plus* v.8.4 software [62] using the baseline, post-intervention, and one-month follow-up data. The originally proposed data analytic strategy [50] entailed the use of multilevel analysis clustered by PE group (i.e., one or more PE classes taught by one teacher) to investigate the effect of the intervention on TCM constructs over time. However, several impediments to this strategy were encountered during preliminary analyses and processing of the data. Due to the relatively low sample size and the complexity of the full TCM, which caused the number of parameters (*n* = 93) to greatly exceed the number of clusters (*n* = 29), the model produced a non-identification error. Constraining residual covariances to zero and reducing non-essential paths only reduced the parameters by 37, so the error remained. We further tested a simplified version of the TCM, which only included perceived autonomy support, the motivation variables, and physical activity. However, this model lacked explanatory value and exhibited poor fit with the data. We therefore opted for traditional path analysis without clustering the data by group. However, as it was possible that variance in the outcome variables may be attributable to group-level variation, we calculated the intraclass correlation coefficient (ICC) for each model construct, which tests the relative contribution of variance in the outcome variable attributable to students in the same group (often referred to as first level variance) and variance attributable to differences between groups (referred to as second level variance) [63]. The ICC ranged from 0 to 1.00, with larger estimates indicating that the PE group to which participants belong contributes substantively to variance in the data [64]. Conversely, smaller values for the ICC suggests there is little variation in the outcome between students sharing the same group, thereby suggesting that the PE group likely has a relatively trivial effect on the differences observed in outcome variables. At post-intervention and at 1 month, respectively, the ICC was 0.003 and 0.001 for physical activity, 0.179 and 0.186 for perceived autonomy support, 0.060 and 0.109 for autonomous motivation in PE, 0.015 and 0.054 for autonomous motivation in leisure time, 0.040 and 0.039 for attitudes, 0.039 and 0.029 for subjective norms, 0.068 and 0.050 for perceived behavioral control, and 0.049 and 0.028 for intentions. Based on the ICC values, we concluded that it was unlikely that the PE group to which students belonged contributed substantially to variance in leisure-time physical activity and the majority of the outcome variables. PE group had the greatest effect on perceived autonomy support and autonomous motivation in PE, although effect sizes were still small.

We tested premises of the TCM using two path analyses: one model predicting physical activity at post-intervention and one model predicting physical activity at one-month follow-up, with students’ leisure-time physical activity as the primary dependent variable, the intervention condition as a dummy-coded independent variable (0 = waitlist control group, 1 = intervention group), and the psychological variables (perceived autonomy support from teachers, autonomous motivation in PE and in leisure time, attitudes, subjective norms, perceived behavioral control, and intentions) as simultaneous predictors. Both models were also regressed on age (continuous variable) and gender (1 = boy, 2 = girl). Given the number of variables and the complexity of the model, we opted not to use an autoregressive longitudinal path analytic model. Instead, change in model constructs was estimated using residual change scores, which is a useful means to control for change while minimizing parameterization [65]. At the post-intervention occasion, residualized change scores of psychological constructs were computed by regressing the score for each construct taken at post-intervention on its score taken at baseline along with the baseline measure of physical activity behavior. For physical activity, the residualized change score was computed by regressing the score for the physical activity variable taken at post-intervention on its score taken at baseline. At the one-month follow-up occasion, residualized change scores of psychological constructs were computed by regressing the score for each construct taken at 1 month on its scores taken at baseline and at post-intervention along with the baseline measure of physical activity behavior. For physical activity, the residualized change score was computed by regressing the score for the physical activity variable taken at the one-month follow-up on its scores taken at baseline and at post-intervention.

Models were implemented using a robust maximum likelihood estimator. Model fit was evaluated using the model chi-square (χ^2^), the comparative fit index (CFI), the Tucker-Lewis index (TLI), the standardized root mean square of the residuals (SRMR), and the root mean square error of approximation (RMSEA). A non-significant chi-square, a CFI value that approaches or exceeds .95, a TLI value that approaches or exceeds .95, a SRMR value of less than .08, and a RMSEA value of .06 or lower, indicate good fit of the model with the data [66]. Model effects were expressed as standardized parameter estimates and direct effect sizes were interpreted as follows: small ≥.20, medium ≥.50, and large ≥.80 [67]. Effect size estimates for indirect effects were less straightforward to interpret because these comprise products of standardized parameter estimates. In this case, we used Seaton et al.’s [68] suggestion that an indirect effect size based on standardized parameter estimates of >.075 represents a non-trivial effect.

For completion, we also conducted a series of 2 (group: intervention group vs. control group) × 3 (time: baseline vs. post-intervention vs. one-month follow-up) mixed-model ANOVAs, with repeated measures on the time factor, to investigate changes in teachers’ self-reported provision of autonomy support and controlling behaviors, and students’ self-reported perceived autonomy support and participation in leisure-time physical activity following the intervention. Statistical significance was determined by *p* < .05. We did not adjust *p*-values for multiple testing. Percentage of missing data for the psychological variables was low (*M* = 0.6%; range 0.0 to 4.3%) and the data were missing completely at random (Little’s MCAR test Chi-square = 6754.139, *df* = 6573, *p* = .058). Missing data were estimated using the full information maximum likelihood estimation (FIML) method. The IPAQ data were processed according to established guidelines^3^ [69].

## Results

### Preliminary analyses

**Fig. 2:**
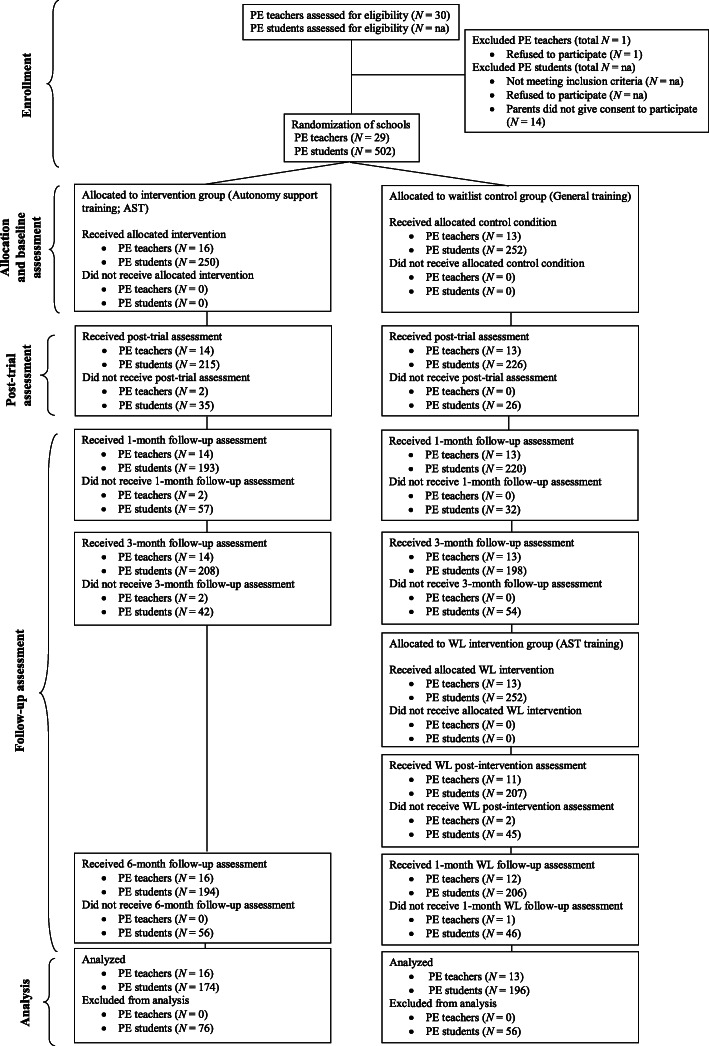
PETALS Intervention Participant Flow Diagram

Out of the 502 students and 29 PE teachers that agreed to participate in the study (85.52% consent rate), 250 students and 16 teachers were randomized to the autonomy support intervention condition, and 252 students and 13 teachers were randomized to the waitlist control condition. Participant flow through the study is presented in Fig. 2. Of the initial 502 participants, 132 were excluded for incomplete data or for providing incorrect responses on the IPAQ. The baseline sample therefore comprised 370 students, 174 in the intervention group (48.28% female; *M* age = 14.69, *SD* = 0.62 years) and 196 in the waitlist control group (53.06% female; *M* age = 14.34, *SD* = 0.71 years). Full descriptive statistics of sample characteristics are presented in Table 1. At baseline, boys were, on average, more active than girls, *t* (368) = 2.616, *p* = .009, but reported lower perceived autonomy support, *t* (368) = − 2.228, *p* = .026. There were no differences in leisure-time physical activity or perceived autonomy support at baseline based on age. Participants in the waitlist control group were, on average, younger, *t* (368) = 4.915, *p* < .001, and reported higher walking MET-minutes at baseline, *t* (368) = − 3.206, *p* = .001, than participants in the intervention group. No differences across groups were observed in gender ratio, χ^2^(1) = 0.845, *p* = .358, or levels of perceived autonomy support at baseline, *t* (368) = 0.949, *p* = .343. With regards to teachers, those assigned to the intervention condition reported a lower perceived provision of controlling behaviors at baseline, *t* (27) = 2.266, *p* = .032, but no differences across groups were observed in teachers’ age, *t* (27) = − 0.434, *p* = .668, teaching experience, *t* (27) = − 0.244, *p* = .809, or perceived provision of autonomy support at baseline, *t* (27) = 0.059, *p* = .953. We also examined differences in baseline characteristics between participants who had six-month follow-up data and a maximum of two non-consecutive missing measurement points (completers; *n* = 296) and participants who did not fulfil these requirements (non-completers; *n* = 74; see Table S3, Appendix E, supplementary materials). There were no significant differences between completers and non-completers in age, gender, or baseline physical activity (*p*s > .05). Finally, a one-way MANOVA was used to test for differences in TCM constructs between completers and non-completers (i.e., perceived autonomy support, autonomous motivation in PE and leisure time, subjective norms, perceived behavioral control, attitudes, and intentions). The result was not significant, *F* (7, 362) = 0.777, *p* = .607; Wilks’ Λ = .985; partial η^2^ = .015, indicating that completers and non-completers did not differ on TCM constructs at baseline. Baseline descriptive and reliability coefficients for study variables and correlations between study variables are displayed in Tables S4 and S5, respectively (see Appendix F, supplementary materials). We also conducted correlational analysis between total self-reported physical activity and physical activity measured using accelerometry to provide concurrent validity data for the IPAQ. Correlations were significant at baseline (*n* = 77; *r* = .245, *p* = .032) and post-intervention (*n* = 56; *r* = .290, *p* = .030).

**Table 1:** Differences Between Intervention and Waitlist Control Group Participants on Baseline Characteristics

	Total Sample	Intervention	Waitlist	Sig.
***Students***	***N*** **=** **370**	***N*** **=** **174**	***N*** **=** **196**	
Age, years	14.50 (0.69)	14.69 (0.62)	14.34 (0.71)	<.001
Gender, *n* (%)				.358
Boy	182 (49.19)	90 (51.72)	92 (46.94)	
Girl	188 (50.81)	84 (48.28)	104 (53.06)	
PA, minutes/week				
Total PA	1322.75 (822.09)	1244.46 (826.80)	1392.25 (813.68)	.046
Vigorous PA	411.70 (344.87)	415.32 (354.90)	408.48 (336.59)	.970
Moderate PA	437.74 (347.10)	427.41 (332.27)	446.92 (360.34)	.823
Walking	473.31 (409.73)	401.73 (389.34)	536.85 (417.81)	.001
PA, MET-minutes/week				
Total PA	6606.47 (4206.17)	6357.89 (4288.37)	6827.14 (4130.26)	.163
Vigorous PA	3293.58 (2758.96)	3322.53 (2839.21)	3267.88 (2692.75)	.970
Moderate PA	1750.98 (1388.38)	1709.66 (1329.09)	1787.66 (1441.38)	.823
Walking	1561.91 (1352.10)	1325.71 (1284.82)	1771.60 (1378.76)	.001
Perceived autonomy support	5.68 (0.89)	5.73 (0.82)	5.64 (0.95)	.343
***Teachers***	***N*** **=** **29**	***N*** **=** **16**	***N*** **=** **13**	
Age, years	42.83 (9.53)	42.13 (9.77)	43.69 (9.54)	.668
Teaching experience, years	15.21 (9.48)	14.81 (10.09)	15.69 (9.04)	.809
Provision of autonomy support	5.39 (0.55)	5.40 (0.55)	5.39 (0.57)	.953
Provision of control	3.77 (0.94)	4.10 (0.84)	3.36 (0.93)	.032

*Note*. *PA* Physical activity. Data presented as means (standard deviations) unless otherwise noted. Differences compared using the Chi-square and *t*-tests. Physical activity MET-minutes were calculated by multiplying days of doing each type of physical activity × minutes spent doing each type of physical activity × MET value. MET values were set at 3.3 for walking, 4.0 for MPA, and 8.0 for VPA [67]

### Main analyses

#### Change in teachers’ provision of autonomy support and controlling behaviors

In terms of teachers’ perceived provision of autonomy support, mixed-model ANOVAs revealed no time, *F* (1.604, 40.091) = 0.992, *p* = .364, η_p_^2^ = .038, group, *F* (1, 25) = 0.225, *p* = .639, η_p_^2^ = .009, or time × group interaction effects, *F* (1.604, 40.091) = 1.201, *p* = .303, η_p_^2^ = .046. There were also no effects of time, *F* (2, 50) = 1.871, *p* = .165, η_p_^2^ = .070, group, *F* (1, 25) = 1.979, *p* = .172, η_p_^2^ = .073, or time × group interaction, *F* (2, 50) = 1.598, *p* = .212, η_p_^2^ = .060, on teachers’ perceived provision of controlling behaviors.

#### Change in students’ perceived autonomy support and engagement in leisure-time physical activity

Mixed-model ANOVAs revealed a statistically significant effect of time, *F* (2, 736) = 5.569, *p* = .004, η_p_^2^ = .015, on students’ perceived autonomy support with a small effect size, but no group, *F* (1, 368) = 0.164, *p* = .686, η_p_^2^ = .000, or time × group interaction effects, *F* (2, 736) = 2.114, *p* = .021, η_p_^2^ = .006. Pairwise comparisons indicated that perceived autonomy support decreased from baseline to one-month follow-up in both groups (*p* = .004). In terms of physical activity behavior, there was also a statistically significant effect of time, *F* (1.813, 449.503) = 7.229, *p* = .001, η_p_^2^ = .028, with a small effect size. Overall, self-reported physical activity decreased from 6606.47 (4206.17) MET-minutes at baseline to 5589.14 (3835.10) MET-minutes at post-intervention and increased slightly to 5629.77 (3939.37) MET-minutes at one-month follow-up.^4^ There was no effect of group, *F* (1, 248) = 0.312, *p* = .577, η_p_^2^ = .001, or time × group interaction, *F* (1.813, 449.503) = 0.152, *p* = .838, η_p_^2^ = .001.

#### Path analytic models

**Fig. 3:**
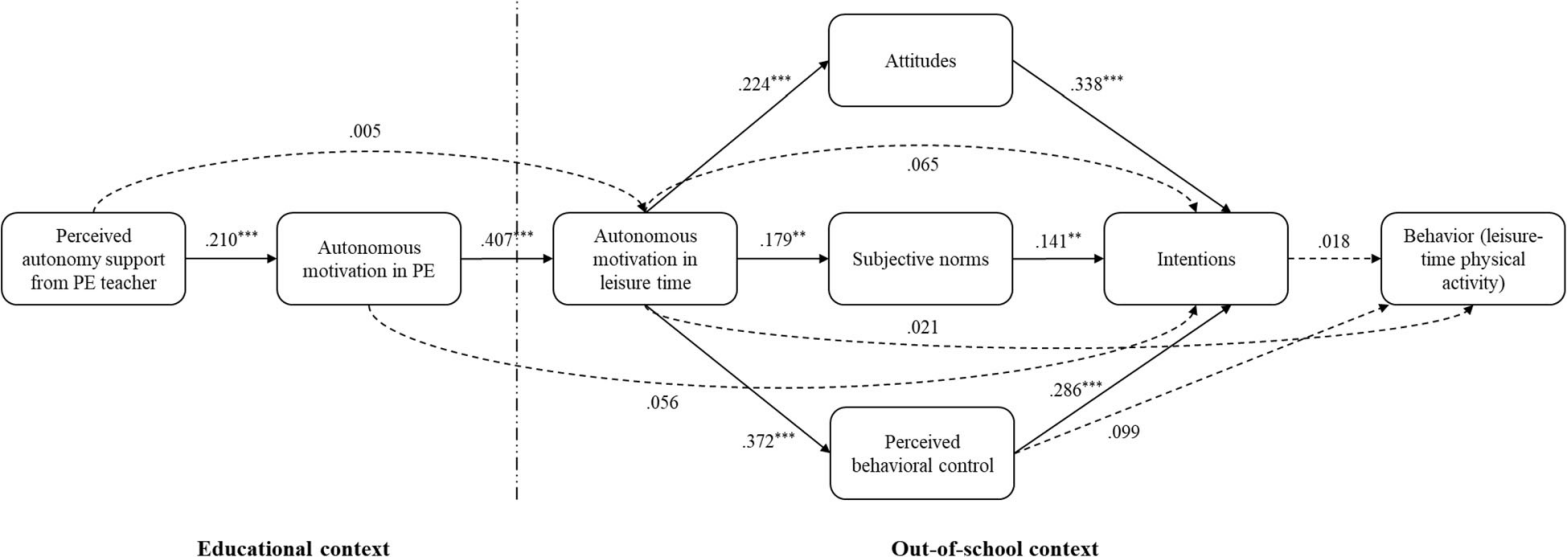
Standardized Parameter Estimates From Path Analysis of the Trans-Contextual Model at Post-Intervention*. Note*. PE = Physical education. Solid lines indicate statistically significant effects among the model variables and dashed lines indicate non-significant effects. There was no effect of the intervention on intentions (β = .051, CI_95_ [−.026, .127], *p* = .278) or physical activity behavior (β = −.009, CI_95_ [−.110, .093], *p* = .888). Hypothesized correlations among study variables not presented: Attitudes ↔ subjective norms *r* = .116, *p* = .047; attitudes ↔ perceived behavioral control *r* = .382, *p* < .001; subjective norms ↔ perceived behavioral control *r* = .255, *p* < .001. ^*^p < .05. ^**^p < .01. ^***^p < .001

**Fig. 4:**
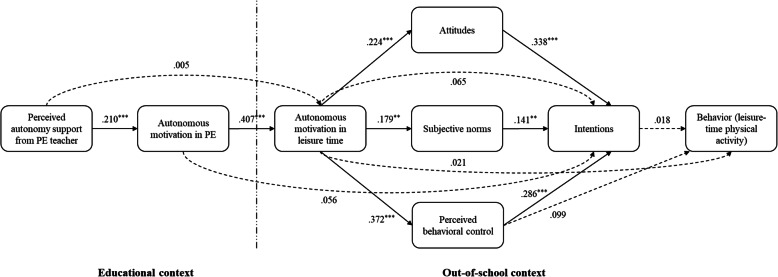
Standardized Parameter Estimates From Path Analysis of the Trans-Contextual Model at One-Month Follow-up. Note. PE = Physical education. Solid lines indicate statistically significant effects among the model variables and dashed lines indicate non-significant effects. There was no effect of the intervention on intentions (β = −.070, CI_95_ [−.149, .008], *p* = .141) or physical activity behavior (β = .015, CI_95_ [−.093, .122], *p* = .821). Hypothesized correlations among study variables not presented: Attitudes ↔ subjective norms *r* = .172, *p* = .003; attitudes ↔ perceived behavioral control *r* = .392, *p* < .001; subjective norms ↔ perceived behavioral control *r* = .195, *p* = .001. ^*^p < .05. ^**^p < .01. ^***^p < .001

We estimated path analytic models predicting leisure-time physical activity engagement and TCM constructs in the intervention and control groups using standardized residual change scores to model change over time. We estimated two models, one using residualized change scores from baseline to post-intervention (Model 1) and one using residualized change scores from baseline to one-month follow-up (Model 2). Standardized parameter estimates for model effects are presented in Table 2 and Figs. 3 and 4.

**Table 2:** Parameter Estimates (β) with 95% Confidence Intervals for Hypothesized Effects from the Structural Equation Model of the Trans-Contextual Model at Post-Intervention and One-Month Follow-up

Independent Variable	Dependent Variable	Mediator	Post-Intervention				One-Month Follow-up			
			β	95% CI		*p*	β	95% CI		*p*
				LL	UL			LL	UL	
*Direct effects*										
PAS	Aut. mot. (PE)		.336^***^	.250	.423	.000	.210^***^	.114	.306	.000
PAS	Aut. mot. (LT)		.201^**^	.105	.296	.001	.005	−.088	.097	.932
Aut. mot. (PE)	Aut. mot. (LT)		.244^***^	.150	.338	.000	.407^***^	.325	.490	.000
Aut. mot. (PE)	Intentions		.058	−.021	.137	.227	.056	−.028	.139	.273
Aut. mot. (LT)	Attitudes		.193^**^	.100	.287	.001	.224^***^	.130	.318	.000
Aut. mot. (LT)	Sub. norms		.155^**^	.062	.248	.006	.179^**^	.082	.275	.002
Aut. mot. (LT)	PBC		.234^***^	.142	.325	.000	.372^***^	.285	.459	.000
Aut. mot. (LT)	Intentions		.291^***^	.213	.369	.000	.065	−.026	.156	.241
Aut. mot. (LT)	Physical activity		.184^**^	.076	.293	.005	.021	−.099	.142	.771
Attitudes	Intentions		.132^**^	.050	.213	.008	.338^***^	.253	.423	.000
Sub. norms	Intentions		.220^***^	.142	.298	.000	.141^**^	.061	.221	.004
PBC	Intentions		.307^***^	.224	.390	.000	.286^***^	.197	.376	.000
PBC	Physical activity		−.038	−.157	.081	.598	.099	−.031	.228	.212
Intentions	Physical activity		.012	−.116	.139	.879	.018	−.104	.140	.806
Age	Intentions		.019	−.057	.096	.679	.019	−.060	.097	.699
Age	Physical activity		−.051	−.152	.050	.402	.093	−.016	.202	.162
Gender	Intentions		.024	−.052	.100	.605	.006	−.072	.084	.900
Gender	Physical activity		.006	−.092	.103	.926	−.064	−.168	.040	.313
Allocation	Intentions		.051	−.026	.127	.278	−.070	−.149	.008	.141
Allocation	Physical activity		−.009	−.110	.093	.888	.015	−.093	.122	.821
*Indirect effects*										
PAS	Aut. mot. (LT)	Aut. mot. (PE)	.082^**^	.044	.120	.001	.085^**^	.042	.129	.001
Aut. mot. (LT)	Intentions	Attitudes	.025^*^	.005	.045	.036	.076^**^	.039	.113	.001
Aut. mot. (LT)	Intentions	Sub. norms	.034^*^	.011	.058	.017	.025^*^	.005	.045	.035
Aut. mot. (LT)	Intentions	PBC	.072^***^	.038	.106	.000	.107^***^	.065	.149	.000
Aut. mot. (PE)	Intentions	Aut. mot. (LT) Attitudes	.006	.001	.012	.061	.031^**^	.014	.047	.002
Aut. mot. (PE)	Intentions	Aut. mot. (LT) Sub. norms	.008^*^	.002	.015	.038	.010^*^	.002	.019	.043
Aut. mot. (PE)	Intentions	Aut. mot. (LT) PBC	.018^**^	.007	.028	.007	.043^***^	.024	.063	.000
Aut. mot. (PE)	Physical activity	Aut. mot. (LT) Attitudes Intentions	.000	−.001	.001	.880	.001	−.003	.004	.806
Aut. mot. (PE)	Physical activity	Aut. mot. (LT) Sub. norms Intentions	.000	−.001	.001	.880	.000	−.001	.001	.807
Aut. mot. (PE)	Physical activity	Aut. mot. (LT) PBC Intentions	.000	−.002	.002	.880	.001	−.005	.006	.806
Aut. mot (LT)	Physical activity	Intentions	.003	−.034	.040	.879	.001	−.007	.009	.810
Aut. mot. (LT)	Physical activity	Attitudes Intentions	.000	−.003	.004	.880	.001	−.008	.011	.806
Aut. mot. (LT)	Physical activity	Sub. norms Intentions	.000	−.004	.005	.880	.000	−.003	.004	.807
Aut. mot. (LT)	Physical activity	PBC Intentions	.001	−.008	.010	.879	.002	−.011	.015	.806
Attitudes	Physical activity	Intentions	.002	−.015	.018	.880	.006	−.035	.048	.806
Sub. norms	Physical activity	Intentions	.003	−.025	.031	.879	.003	−.015	.020	.807
PBC	Physical activity	Intentions	.004	−.035	.043	.879	.005	−.030	.040	.806
*Sums of indirect effects*										
Aut. mot. (LT)	Intentions	Multiple^a^	.131^***^	.084	.178	.000	.207^***^	.148	.267	.000
Aut. mot. (LT)	Physical activity	Multiple^a^	−.004	−.050	.042	.888	.042	−.004	.087	.132
PAS	Physical activity	Multiple^a^	.051^**^	.019	.083	.008	.006	−.006	.018	.413
PAS	Intentions	Multiple^a^	.139^***^	.088	.189	.000	.036	.003	.070	.077
*Total effects*										
PAS	Intentions	Multiple^b^	.139^***^	.088	.189	.000	.036	.003	.070	.077
PAS	Physical activity	Multiple^b^	.051^**^	.019	.083	.008	.006	−.006	.018	.413
Aut. mot. (LT)	Intentions	Multiple^b^	.422^***^	.340	.504	.000	.272^***^	.175	.370	.000
Aut. mot. (LT)	Physical activity	Multiple^b^	.180^**^	.084	.277	.002	.063	−.048	.174	.350
*Correlations*										
Attitudes ↔ Sub. norms			.116^*^	.020	.212	.047	.172^**^	.076	.267	.003
Attitudes ↔ PBC			.382^***^	.299	.466	.000	.392^***^	.308	.475	.000
Sub. norms ↔ PBC			.255^***^	.164	.346	.000	.195^**^	.100	.290	.001

*Note*. ^a^Mediators for this effect included effects of the predictor on the outcome through multiple mediators. ^b^Mediators for this effect included effects of the predictor on the outcome through multiple mediators along with the direct effect of the predictor variable on the outcome. β = Standardized parameter estimate; 95% CI = 95% confidence interval of path coefficient; PAS = Perceived autonomy support; Aut. mot. (PE) = Autonomous motivation (in physical education); Aut. mot. (LT) = Autonomous motivation (in leisure time); PBC = Perceived behavioral control; Sub. norms = Subjective norms. ^*^p < .05. ^**^p < .01. ^***^p < .001

Focusing on Model 1, the model exhibited acceptable fit with the data according to the adopted goodness-of-fit indices, χ^2^(11) = 22.097, *p* = .024, RMSEA = .058, CI_90_ [.021, .093], *p* = .312, CFI = .967, TLI = .842, SRMR = .034. Consistent with ANOVA results, there were no effects of the intervention on baseline-adjusted TCM constructs or physical activity behavior at post-intervention. However, we found direct effects of perceived autonomy support on autonomous motivation in PE (β = .336, CI_95_ [.250, .423], *p* < .001), autonomous motivation in PE on autonomous motivation in leisure time (β = .244, CI_95_ [.150, .338], *p* < .001), and autonomous motivation in leisure time on attitudes (β = .193, CI_95_ [.100, .287], *p* = .001), subjective norms (β = .155, CI_95_ [.062, .248], *p* = .006), and perceived behavioral control (β = .234, CI_95_ [.142, .325], *p* < .001). Subjective norms (β = .220, CI_95_ [.142, .298], *p* < .001), attitudes (β = .132, CI_95_ [.050, .213], *p* = .008), perceived behavioral control (β = .307, CI_95_ [.224, .390], *p* < .001), and autonomous motivation in leisure time (β = .291, CI_95_ [.213, .369], *p* < .001) predicted intentions. Only autonomous motivation in leisure time significantly predicted physical activity at post-intervention (β = .184, CI_95_ [.076, .293], *p* = .005). All effects were small in size. We also found indirect effects of perceived autonomy support on autonomous motivation in leisure time mediated by autonomous motivation in PE (β = .082, CI_95_ [.044, .120], *p* < .001). There was an indirect effect of autonomous motivation in PE on intentions (β = .103, CI_95_ [.058, .148], *p* < .001) and physical activity (β = .045, CI_95_ [.015, .074], *p* = .013). There were also indirect effects of autonomous motivation in leisure time on intentions mediated by subjective norms (β = .034, CI_95_ [.011, .058], *p* = .017), attitudes (β = .025, CI_95_ [.005, .045], *p* = .036), and perceived behavioral control (β = .072, CI_95_ [.038, .106], *p* < .001). There were significant total effects of perceived autonomy support on intentions (β = .139, CI_95_ [.088, .189], *p* < .001) and physical activity (β = .051, CI_95_ [.019, .083], *p* = .008).

Focusing on Model 2, the model exhibited acceptable fit with the data according to the adopted goodness-of-fit indices, χ^2^(11) = 10.993, *p* = .444, RMSEA = .000 CI_90_ [.000, .061], *p* = .877, CFI = 1.000, TLI = 1.000, SRMR = .026. We found no effects of the intervention on TCM constructs or physical activity behavior at 1 month. However, confirming findings from Model 1, we found direct effects of perceived autonomy support on autonomous motivation in PE (β = .210, CI_95_ [.114, .306], *p* < .001), autonomous motivation in PE on autonomous motivation in leisure time (β = .407, CI_95_ [.325, .490], *p* < .001), and autonomous motivation in leisure time on attitudes (β = .224, CI_95_ [.130, .318], *p* < .001), subjective norms (β = .179, CI_95_ [.082, .275], *p* = .002), and perceived behavioral control (β = .372, CI_95_ [.285, .459], *p* < .001). Subjective norms (β = .141, CI_95_ [.061, .221], *p* = .004), attitudes (β = .338, CI_95_ [.253, .423], *p* < .001), and perceived behavioral control (β = .286, CI_95_ [.197, .376], *p* < .001) predicted intentions. All effects were small in size. We also found indirect effects of perceived autonomy support on autonomous motivation in leisure time mediated by autonomous motivation in PE (β = .085, CI_95_ [.042, .129], *p* = .001). There was an indirect effect of autonomous motivation in PE on intentions (β = .111, CI_95_ [.064, .157], *p* < .001). There were also indirect effects of autonomous motivation in leisure time on intentions mediated by subjective norms (β = .025, CI_95_ [.005, .045], *p* = .035), attitudes (β = .076, CI_95_ [.039, .113], *p* = .001), and perceived behavioral control (β = .107, CI_95_ [.065, .149], *p* < .001). We found no total effects of perceived autonomy support on intentions (β = .036, CI_95_ [.003, .070], *p* = .077) or physical activity (β = .006, CI_95_ [−.006, .018], *p* = .413) at 1 month. The null findings for the intervention precluded examination of the indirect effects of the intervention on physical activity participation through the mediation of the TCM constructs in both models.

### Ancillary analyses

Given the null effects for the intervention, we conducted follow-up analyses to investigate whether effects of the intervention depended on baseline levels of activity among the participating students. We therefore conducted a split-plot ANOVA to assess the impact of baseline physical activity level on physical activity change over time following the intervention. Participants were categorized as having either a low, moderate, or high level of physical activity based on computed percentiles. There were no main effects of time, *F* (1.799, 444.233) = 0.346, *p* = .685, η_p_^2^ = .001, group, *F* (1, 247) = 0.122, *p* = .727, η_p_^2^ = .000, or time × group interaction effect, *F* (1.799, 444.233) = 0.108, *p* = .878, η_p_^2^ = .000, on physical activity. Importantly, although there was a trend towards a time × baseline physical activity level interaction effect on physical activity participation, *F* (1.799, 444.233) = 3.049, *p* = .054, η_p_^2^ = .012, implying that physical activity decreased only in participants who had high or moderate baseline levels of physical activity, it did not meet the conventional level of statistical significance and the effect size was small.

## Discussion

The purpose of the current research was to examine the efficacy of a PE teacher-delivered autonomy supportive intervention, based on the TCM, for increasing leisure-time physical activity engagement in lower secondary school students. Effects of the intervention were evaluated through changes in post-intervention follow-up measures (post-intervention after the implementation period and at one-month follow-up) of students’ leisure-time physical activity and TCM model variables relative to pre-trial baseline measures. The research also aimed to examine whether intervention effects on changes in leisure-time physical activity participation were mediated by constructs from the TCM.

Contrary to our predictions, we found no effect of the intervention on TCM variables or physical activity behavior at post-intervention or one-month follow-up data collection occasions. These null effects meant that proposed indirect effects of the intervention on physical activity participation through the TCM constructs were also no different from zero. However, we found support for multiple propositions of the TCM. At post-intervention, we found direct effects of perceived autonomy support on autonomous motivation in PE, autonomous motivation in PE on autonomous motivation in leisure time, and autonomous motivation in leisure time on attitudes, subjective norms, and perceived behavioral control. Subjective norms, attitudes, perceived behavioral control, and autonomous motivation in leisure time predicted intentions. Autonomous motivation in leisure time also predicted physical activity at post-intervention. In terms of indirect effects, perceived autonomy support predicted autonomous motivation in leisure time mediated by autonomous motivation in PE. Autonomous motivation in PE indirectly predicted intentions and physical activity behavior through autonomous motivation in leisure time and the belief-based constructs. Autonomous motivation in leisure time predicted intentions mediated by subjective norms, attitudes, and perceived behavioral control. Perceived autonomy support had significant total effects on intentions and physical activity behavior, consistent with previous studies and reviews [42, 70, 71]. Results from one-month follow-up data corroborated the results observed at post-intervention, with the exception that we found no direct effect of autonomous motivation in leisure time on physical activity behavior or total effect of perceived autonomy support on intentions or physical activity behavior.

Our study supports findings from recent research that shows that multiple predictions of the TCM hold when modeling change in constructs over time [43]. However, contrary to our hypotheses, we found no support for the efficacy of the autonomy-supportive intervention at increasing students’ leisure-time physical activity and related constructs from the TCM. We present several potential interpretations for these findings. One explanation is that students already reported relatively high levels of perceived autonomy support and autonomous motivation at baseline. Examination of mean levels for the perceived autonomy support and autonomous motivation constructs at baseline indicated that mean values for these scales were extremely high (> 5.50 on a 7-point scale), which suggests that leeway for improvement was modest. In addition, students in the current study already reported being highly active in their leisure time prior to the intervention and the majority met or exceeded the recommended national guidelines of at least 1 to 2 h of activity daily, in contrast to national data for physical activity [8]. These ‘ceiling’ effects in outcome variables suggest that the scope for change was relatively small. This also points to the fact that the intervention strategies of providing additional autonomy support may not have had much impact on students who were already autonomously motivated, viewed their teachers as autonomy supportive, and were active in their leisure time. Taken together, it seems that students had little scope for improvement and an autonomy supportive intervention may have had little impact on already high perceptions of autonomy.

It is also important to note that teachers likewise reported relatively high levels of perceived provision of autonomy support and low levels of provision of controlling behaviors at baseline. As such, it seemed that teachers may already have been highly proficient in applying autonomy supportive teaching and, therefore, had very little scope to become more autonomy supportive. This issue was corroborated by comments made by external stakeholders in the protocol phase of this study [50], who mentioned that Finnish PE teachers tended to already be relatively autonomy supportive, but also attested that there was considerable variation. Autonomy supportive teaching is currently emphasized both in the Finnish national PE curriculum and in the Finnish PE teacher-training curriculum [72]. It is plausible that the current intervention may have greater efficacy in other contexts and national groups where autonomy support is lacking and is not part of PE teacher training [44].

Although there were no changes in leisure-time physical activity at follow-up as a result of the intervention, baseline physical activity was a substantive predictor of physical activity engagement at follow-up. This suggests that leisure-time physical activity was affected by past physical activity engagement and experience, consistent with previous research demonstrating pervasive effects for past behavior in the prediction of future participation [73–75]. Furthermore, such research indicates that past behavior effects attenuate the strength of effects of other social cognition and motivational determinants, which may also include effects of techniques targeting change in these constructs, as in the current intervention. It is also plausible that other environmental and social variables were more salient determinants of students’ physical activity behavior in the present study than the psychological determinants identified in the current model. Research based on ecological determinants [76, 77] and socio-structural predictors [78, 79] of physical activity behavior illustrate the myriad of potential determinants of children’s leisure-time physical activity engagement, and a focus on psychological determinants alone may have neglected other potential predictors. As an example, students’ activity levels may have been influenced by environmental factors, such as access to sport clubs, ability to walk to school, and taking part in organized physical activity in their leisure time. As such, these factors may have been stronger determinants of physical activity than the current intervention, and were unlikely to change over the duration of the study. There is a need to augment models such as the TCM to include these ecological and socio-structural variables, which have proven to be important additional determinants of health behavior [76, 79, 80]. Indeed, overall, in developing interventions to a particular target group, it is useful to assess what needs and deficits there may be, and then select theory and match the intervention strategies according to this ‘needs assessment’ [81]; if levels of motivation and autonomy support already were high in this target group, perhaps the TCM was not the best suited theory to use as the (sole) basis of the intervention. As such, future studies may consider selecting teachers with lower levels of autonomy support at baseline, and recruiting samples of students with lower levels of perceived autonomy support, autonomous motivation, and leisure-time physical activity at baseline that may be more representative of adolescents at this age.

Furthermore, the intervention exclusively targeted change in the psychological determinants of children’s leisure-time physical activity, specifically participants’ perceived autonomy support from PE teachers and autonomous motivation by changing the autonomy supportive behaviors displayed by teachers. This means that the intervention did not target other potentially influential determinants of physical activity proposed in the TCM. For example, research has indicated that perceived autonomy support from parents and peers, as well as peer norms, are particularly important determinants of adolescents’ physical activity and could be highly salient predictors, especially given the ceiling effects of perceived autonomy support from PE teachers in the current sample (e.g., [30, 40]). These findings suggest that including additional training components of the autonomy supportive training program targeting change in autonomy support from these other social agents may have resulted in larger effects on leisure-time physical activity in the current sample.

Another potential factor that may have affected current findings is the intensiveness of the intervention. The autonomy support training program comprised an intensive two-week intervention, which provided teachers with comprehensive and detailed instruction on the application of autonomy-supportive teaching techniques [28, 45]. While this was congruent with the duration and content of autonomy supportive interventions developed elsewhere that have been shown to be efficacious, the intervention was highly intensive. Informal feedback from the teachers indicated that they were satisfied with the program and they had ample time during the program to demonstrate their skills. Nevertheless, the intensive structure and sheer amount of information delivered in a very short period may have meant that teachers did not have the opportunity to fully learn and assimilate the suggested behaviors. Previous research has shown that teacher’s beliefs about the ease of implementing an autonomy supportive intervention program is important in influencing post-intervention increases in provision of autonomy support [82]. The teacher-training phase of the PETALS intervention was relatively short, compared to previous studies adopting autonomy supportive intervention programs (e.g., [44, 51]), and no booster sessions were provided in the implementation phase. It is possible that such follow-up sessions would have been beneficial to ensure that the teachers fully understood the intervention and how to implement it within their PE lessons. The excess of information may have meant that teachers could not fully implement the intervention behaviors and therefore reported very little change, the high extant levels of autonomy support notwithstanding.

Although the intervention was not shown to be efficacious in affecting change in leisure-time physical activity or the candidate mediators from the TCM, at follow up, we did find support for the constructs that were effective in predicting change in the TCM constructs over time, consistent with previous research [43, 83]. Specifically, it seems that the TCM in the current context is effective in explaining variance in autonomous motivation toward physical activity in both PE and leisure-time contexts from perceived autonomy support, the immediate determinants of intention, and intentions, supporting key predictions in terms of the TCM motivational sequence. However, the model was not effective in explaining change in leisure-time physical activity, with past behavior, that is, activity at baseline, the only predictor. These findings support the capacity of the model in determining leisure-time physical activity intentions, but not behavior. This is a fundamental problem for a model that presents itself as one that seeks to determine behavior. There may be sample-specific determinants that were not accounted for in the current model. Given that past behavior, the sole predictor of leisure-time physical activity in the current study, is purported to represent habitual influences, it may be that the social cognition and motivational determinants in the TCM have little relevance in the present sample because students’ physical activity behavior is habitual. This means that consideration of beliefs and motives is less relevant than automatically-activated behavioral responses initiated by cues or prompts in the environment [84, 85], or deep-seated attitudes or beliefs that are implicitly held and not captured by the current measures [86, 87]. Future research may consider incorporating measures of these constructs into the TCM, as has been done in other integrated models.

### Strengths, limitations, and recommendations for future research

The present study should be considered in light of its multiple strengths, but also some salient limitations. Strengths of the study include: (1) a focus on the determinants of lower secondary school students’ leisure-time physical activity, which is a priority area of research, as physical activity tends to decrease during adolescence; (2) a basis in extant theory that outlines the mechanisms by which the proposed intervention affects change in the target outcome variables, leisure-time physical activity behavior and TCM constructs; (3) the adoption of rigorously-developed theory-based intervention and materials that align with the targeted TCM constructs; (4) the use of an appropriately-powered cluster randomized waitlist-controlled design; and (5) high teacher engagement and attendance in the intervention training program.

However, several limitations should also be noted that may affect interpretation of the findings and the extent to which they can be generalized, as well as for consideration in future research. A key limitation was the exclusive reliance on self-report measures, particularly the primary outcome measure of leisure-time physical activity engagement. Although we collected accelerometer data to provide concurrent validity for the IPAQ, correlations between self-reported and objectively measured physical activity data were modest. A further issue is that there may have been a lack of correspondence between measures of the social cognition predictors from the TCM and the measure of physical activity. TCM measures were designed to focus on high intensity moderate-to-vigorous physical activity (e.g., “I intend to do sports and/or vigorous physical activities …” ), while the IPAQ provides estimates based on physical activities from different intensities including light, moderate, and vigorous activities. This lack of correspondence may have introduced additional error variance in the predictions and reduced the capacity of the intervention and TCM constructs in determining behavior.

Additionally, the causal effects of the TCM are inferred from the theory and not the data [37]. Although the current study employed an intervention design, it only targeted perceived autonomy support and we found no change in perceived autonomy support in either group. As such, we could not establish whether a change in autonomy support would have a causal effect on autonomous motivation and, in turn, the theory of planned behavior variables and physical activity behavior. Intervention and experimental study designs are needed to test the effects of manipulating other constructs of the TCM that have been found to have a direct and significant effect on leisure-time physical activity behavior [17]. Further, there are some limitations to inferring individual behavior change mechanisms from between-persons designs and data. For example in testing self-determination theory relationships, between- and within-person analysis approaches may lead to essentially opposite outcomes and conclusions [88]. We therefore recommend future studies to conduct more nuanced, within-individual modeling in evaluating the TCM, in addition to group-level analyses.

Given that physical activity decreases during adolescent years and physical activity started in young age tracks into adulthood, studies investigating ways to support physical activity in adolescent years are important. More research is required to understand the determinants of leisure-time physical activity in this age group, so that more effective interventions can be developed. Future research should aim to explore individual components of autonomy supportive intervention programs, rather than multiple components. This would reduce intervention complexity and the potential information overload, as well as help test whether or not individual components of autonomy supportive intervention programs are effective [45]. This would also allow for a more comprehensive evaluation of intervention effects and mechanisms [18]. In this endeavor, qualitative methods may also shed light on critical components and change mechanisms, to complement findings from quantitative studies (e.g., [89]). Moreover, due to the relatively high level of physical activity among lower secondary school students found in the current study, as well as the fact that Finnish PE teachers tend to report being autonomy supportive in their teaching, the present intervention should be evaluated in other contexts or countries, where autonomy support among teachers may be lower. Similarly, teachers may have been aware from previous training about what constitutes autonomous versus controlled ways of teaching, which could have created a social desirability bias when answering questionnaires. Future studies might consider including alternative or objective measures of teachers’ provision of autonomy support and control during PE.

## Conclusion

The current study was the first to test the efficacy of an intervention aimed specifically at enhancing perceived autonomy support in lower secondary school students on autonomous motivation, beliefs, intentions, and leisure-time physical activity. Contrary to predictions, results indicated that the intervention was not successful at inducing change in physical activity behavior or TCM constructs at post-intervention or at one-month follow-up. It is plausible that because students already reported high levels of perceived autonomy support at baseline, there was no room for change; however, future research is required to test the PETALS intervention in contexts where this is not the case to establish whether the intervention is more successful when lower baseline values of perceived autonomy support are reported. Similarly, the intervention did not take into account other factors that may have been more important for enhancing students’ motivation towards and engagement in leisure-time physical activity, such as parent and peer support. Future iterations of similar interventions should aim to target other social determinants that have been found to be important in this age group for promoting physical activity behavior in leisure time. More research is required to determine whether the TCM retains predictive validity when individual variables are successfully and systematically manipulated through experimental and intervention studies.

## Supplementary information

**Additional file 1: Appendix A Figure S1.** Timeline of the PETALS Trial. **Appendix B Table S1.** Description of the Teacher-Training Program: Content and Matched Behavior Change Techniques for Each Session [90]. **Appendix C Table S2.** Measures, Data Collection Time Points, and Methods in the PETALS Intervention. **Appendix D** Processing of IPAQ Data [91]. **Appendix E Table S3.** Differences Between Completers and Non-Completers on Baseline Characteristics [67]. **Appendix F Table S4.** Descriptive and Reliability Statistics of Key Variables at Baseline [67]. **Table S5.** Pearson’s Correlations Between Key Variables at Baseline. **Appendix G Table S6.** Descriptive Statistics for Trans-Contextual Model Constructs over Time.

## Data Availability

The datasets analyzed for the current study and the supplementary materials including the intervention manual and accompanying resources are available online on the study project site on the Open Science Framework: https://osf.io/s4b2g/.

## Abbreviations

PE: Physical education
PETALS: Physical education teachers’ autonomy-support training to promote leisure-time physical activity in schoolchildren
TCM: Trans-contextual model

## Footnotes

1 This is a deviation from the original study protocol as we originally planned for waitlist control teachers to receive the autonomy support training program *after* the three-month data collection occasion [50]. As such, we used baseline, post-intervention, and one-month follow-up data to analyze differences between groups.

2 We also collected follow-up data at three and 6 months post-intervention, but only report the immediately post-intervention and one-month follow-up data in the current article, as the waitlist control group received the intervention prior to the three-week data collection occasion.

3 Full details on how IPAQ data were handled are available in Appendix D (supplementary materials).

4 Descriptive statistics for all TCM constructs at baseline, post-intervention, and one-month follow-up are provided in Table S6 (Appendix G, supplementary materials).
