# Supplementary material for: Testing a physical education-delivered autonomy supportive intervention to promote leisure-time physical activity in lower secondary school students: the PETALS trial
Source: BMC Public Health. 2020 Sep 22;20:1438. doi: 10.1186/s12889-020-09518-3 (PMC7510083; doi:10.1186/s12889-020-09518-3)
Supplement: Supplementary file 1 — Additional file 1: Appendix A Figure S1. Timeline of the PETALS Trial. Appendix B Table S1. Description of the Teacher-Training Program: Content and Matched Behavior Change Techniques for Each Session [90]. Appendix C Table S2. Measures, Data Collection Time Points, and Methods in the PETALS Intervention. Appendix D Processing of IPAQ Data [91]. Appendix E Table S3. Differences Between Completers and Non-Completers on Baseline Characteristics [67]. Appendix F Table S4. Descriptive and Reliability Statistics of Key Variables at Baseline [67]. Table S5. Pearson’s Correlations Between Key Variables at Baseline. Appendix G Table S6. Descriptive Statistics for Trans-Contextual Model Constructs over Time. [file 12889_2020_9518_MOESM1_ESM.docx]

# Supplementary Materials

# Appendix A


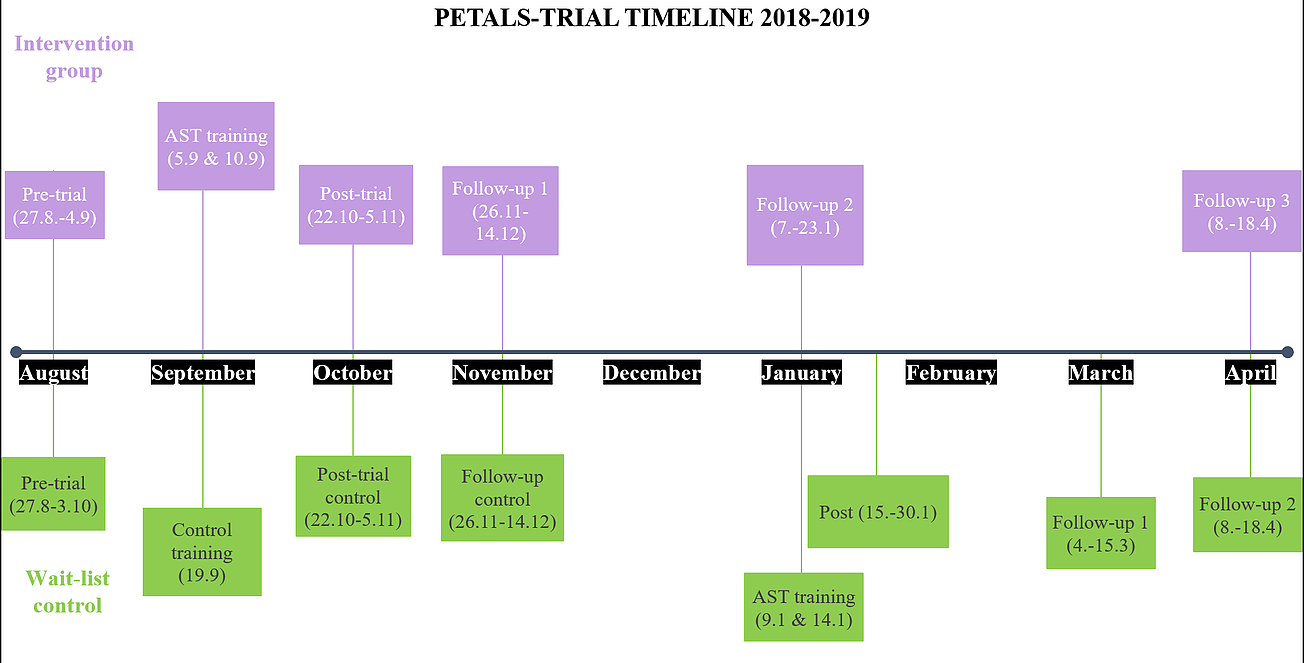


**Figure S1**

*Timeline of the PETALS Trial*

# Appendix B

**Table S1**

*Description of the Teacher-Training Program: Content and Matched Behavior Change Techniques for Each Session*

| Session Topic | Content | Behavior Change Techniques^a^ |
| --- | --- | --- |
| 1. Introduction and added value of the training to teaching practice | Introduction and warm up activities  Information on the added value of the training and expectations  Explore current supportive style and reflection  Why autonomy support matters  Introduction to self-determination theory | Social support (unspecified)  Social support (practical)  Discrepancy between current behavior and goal  Shaping knowledge  Information about social and environmental consequences  Imaginary reward |
| 2. Autonomy supportive techniques: Description and benefits for students and teachers | Basics of the autonomy supportive teaching techniques: Definitions and implementation examples  Using autonomy supportive techniques: Benefits for students and teachers based on previous research results | Demonstration of the behavior  Shaping knowledge  Information about social and environmental consequences  Behavioral practice/rehearsal |
| 3. Use of autonomy supportive techniques to provide instructions | How, when, and why to use autonomy supportive techniques when giving instructions (organizational, technical and tactical)  Taking students’ perspective  Using non-controlling and informational language  Providing rationale  Providing choices  Displaying patience | Information about social and environmental consequences  Instruction on how to perform a behavior  Behavior substitution  Habit reversal  Framing/reframing  Self-monitoring of behavior  Behavioral experiments  Behavioral practice/rehearsal  Graded tasks |
| 4. Use of autonomy supportive techniques to provide feedback, encouragement, and praise | How, when, and why to use autonomy supportive techniques when providing feedback, encouragement, and praise  Using non-controlling and informational language  Taking students’ perspective  Displaying patience | Generalization of a target behavior  Behavior substitution  Habit reversal  Framing/reframing  Information about social and environmental consequences  Instruction on how to perform a behavior  Self-monitoring of behavior  Behavioral experiments  Behavioral practice/rehearsal  Graded tasks |
| 5. Use of autonomy supportive techniques to deal with discipline issues and off-task behaviors | How, when, and why to use autonomy supportive techniques when dealing with discipline issues and off task behaviors  Taking students perspective  Accepting negative affect  Providing rationale  Using non-controlling and informational language  Displaying patience  Providing choices |  |
| 6. Building personalized action plans | Plan changes in own teaching practice: Specific goals and plans for change when giving instructions, provide feedback, and respond to students with low motivation  Identify barriers and problem solving for using the autonomy supportive techniques in every day teaching practice  Development of their individualized infographic poster | Generalization of a target behavior  Goal setting  Action planning (and implementation intention)  Instruction on how to perform a behavior  Prompts/cues  Adding objects to the environment  Pros and cons  Social support unspecified |

*Note*. ^a^From behavior change technique taxonomy (Version 1) [90].

# Appendix C

**Table S2**

*Measures, Data Collection Time Points, and Methods in the PETALS Intervention*

| Assessment | Pre | Post | 1MFU | 3MFU | 6MFU |
| --- | --- | --- | --- | --- | --- |
| *Main outcome* | | | | | |
| Out-of-school physical activity^os^ | SR^1^ | SR^1^ | SR^1^ | SR^1^ | SR^1^ |
| *Secondary outcome* |  |  |  |  |  |
| Accelerometry^is, os^ | PA^1^ | PA^1^ | − | PA^1^ | PA^1^ |
| *Mediating variables* |  |  |  |  |  |
| Perceived autonomy support from PE teacher^is, os^ | SR^1^ | SR^1^ | SR^1^ | SR^1^ | SR^1^ |
| Perceived autonomy support from parents^os^ | SR^1^ | SR^1^ | SR^1^ | SR^1^ | SR^1^ |
| Perceived autonomy support from peers^os^ | SR^1^ | SR^1^ | SR^1^ | SR^1^ | SR^1^ |
| Autonomous motivation^is, os^ | SR^1^ | SR^1^ | SR^1^ | SR^1^ | SR^1^ |
| Controlled motivation^is, os^ | SR^1^ | SR^1^ | SR^1^ | SR^1^ | SR^1^ |
| Amotivation^is, os^ | SR^1^ | SR^1^ | SR^1^ | SR^1^ | SR^1^ |
| Attitudes^os^ | SR^1^ | SR^1^ | SR^1^ | SR^1^ | SR^1^ |
| Subjective norms^os^ | SR^1^ | SR^1^ | SR^1^ | SR^1^ | SR^1^ |
| Perceived behavioral control^os^ | SR^1^ | SR^1^ | SR^1^ | SR^1^ | SR^1^ |
| Intentions^os^ | SR^1^ | SR^1^ | SR^1^ | SR^1^ | SR^1^ |
| *Additional measures* | | | | | |
| Observation of teacher autonomy support | OB^2^ | OB^2^ | − | OB^2^ | OB^2^ |
| Behavioral automaticity | SR^1^ | − | − | − | − |
| Grit | SR^1^ | − | − | − | − |
| Self-discipline | SR^1^ | − | − | − | − |
| *Teacher measures* |  |  |  |  |  |
| Provision of autonomy support | SR^2^ | SR^2^ | SR^2^ | SR^2^ | SR^2^ |
| Provision of control | SR^2^ | SR^2^ | SR^2^ | SR^2^ | SR^2^ |
| *Parenting measures* |  |  |  |  |  |
| Parental affection | SR^1, 3^ | − | − | − | − |
| Parenting behavioral control | SR^1, 3^ | − | − | − | − |
| Parenting psychological control | SR^1, 3^ | − | − | − | − |
| *Demographics* |  |  |  |  |  |
| Age | SR^1, 2, 3^ | − | − | − | − |
| Gender | SR^1, 2, 3^ | − | − | − | − |
| Education | SR^2, 3^ | − | − | − | − |
| Ethnicity | SR^2^ | − | − | − | − |
| School | SR^1, 2^ | − | − | − | − |
| Grade | SR^1^ | − | − | − | − |
| Years of teaching physical education | SR^2^ | − | − | − | − |
| Number of students in teachers’ PE class | SR^2^ | − | − | − | − |
| Socioeconomic status | SR^3^ | − | − | − | − |

*Note*. SR = Self-report measure; OB = Data collected by observation; Pre = Pre-trial data collection occasion; Post = post-intervention data collection occasion; 1MFU = One-month follow-up data collection occasion; 3MFU = Three-month follow-up data collection occasion; 6MFU = Six-month follow-up data collection occasion. For each assessment, the method of data collection is indicated as SR for self-report, OB for observation, and PA for physical activity surveillance; to whom each measure is administered is indicated as ^1^ for students, ^2^ for physical education teachers, and ^3^ for parents; context evaluated by each measure is indicated as ^IS^ for in-school and ^OS^ for out-of-school.

# Appendix D

## Processing of IPAQ Data

Initially, the values from hours or hours and minutes were translated into minutes. Values of 15, 30, 45, 60, or 90 in the ‘hours’ column of the questionnaire were transferred to the ‘minutes’ column. Participants who made mistakes not addressed in the guidelines (e.g., value of 25 hours or missing data for days or time) were excluded. Secondly, when the sum of weekly PA exceeded 6720 minutes (i.e., 16 hours × 60 minutes × 7 days) participants were excluded with the assumption that they were sleeping eight hours a day. Thirdly, truncation (re-coding) of IPAQ scores was performed. Any given activity above three hours was re-coded to three hours (i.e., 180 minutes), permitting a maximum value of 21 hours per activity (three hours × seven days) and 63 hours (i.e., 3780 minutes) of total PA per week. Similarly, any value below 10 minutes was recoded to zero. Finally, due to the skewed distributions associated with the IPAQ, the MET-minute values were log transformed using the log10 + 1 transformation in SPSS to account for zero values. The distribution of the log-transformed MET-minutes has the property of being more normally distributed, in contrast to the raw IPAQ scores [91].

# Appendix E

**Table S3**

*Differences Between Completers and Non-Completers on Baseline Characteristics*

|  | Completers  (*N* = 296) | Non-Completers  (*N* = 74) | Sig. |
| --- | --- | --- | --- |
| *Students* |  |  |  |
| Age, years | 14.05 (0.78) | 14.01 (0.69) | .683 |
| Gender, *n* (%) |  |  | .086 |
| Boy | 139 (46.96) | 43 (58.11) |  |
| Girl | 157 (53.04) | 31 (41.89) |  |
| PA, minutes/week |  |  |  |
| Total PA | 1295.26 (796.83) | 1432.72 (913.81) | .367 |
| Vigorous PA | 405.42 (335.15) | 436.82 (382.73) | .866 |
| Moderate PA | 425.46 (342.85) | 486.89 (361.79) | .157 |
| Walking | 464.38 (401.62) | 509.00 (441.75) | .570 |
| PA, MET-minutes/week |  |  |  |
| Total PA | 6477.62 (4071.30) | 7121.86 (4702.02) | .397 |
| Vigorous PA | 3243.32 (2681.19) | 3494.59 (3061.81) | .866 |
| Moderate PA | 1701.83 (1371.42) | 1947.57 (1447.16) | .157 |
| Walking | 1532.46 (1325.34) | 1679.70 (1457.79) | .570 |
| Perceived autonomy support | 5.69 (0.91) | 5.67 (0.81) | .886 |

*Note*. PA = Physical activity. Completers defined as participants who had six-month follow-up data and a maximum of two non-consecutive missing time points. Data presented as means (standard deviations) unless otherwise noted. Differences compared using the Chi-square and *t*-tests. Physical activity MET-minutes were calculated by multiplying days of doing each type of physical activity × minutes spent doing each type of physical activity × MET value. MET values were set at 3.3 for walking, 4.0 for MPA, and 8.0 for VPA [67].

# Appendix F

**Table S4**

*Descriptive and Reliability Statistics of Key Variables at Baseline*

| Study Variable | Number of scale items | *M* | *SD* | α |
| --- | --- | --- | --- | --- |
| Vigorous intensity physical activity | 2 | 3293.58 | 2758.96 | − |
| Moderate intensity physical activity | 2 | 1750.98 | 1388.38 | − |
| Walking | 2 | 1561.91 | 1352.10 | − |
| Total physical activity | 6 | 6606.47 | 4206.17 | − |
| Perceived autonomy support from PE teacher | 13 | 5.68 | 0.89 | .916 |
| Perceived control from PE teacher | 5 | 4.20 | 1.09 | .782 |
| Perceived autonomy support from parents | 4 | 6.34 | 0.90 | .855 |
| Perceived autonomy support from peers | 4 | 5.26 | 1.28 | .844 |
| Autonomous motivation in PE | 4 | 5.58 | 1.35 | .889 |
| Autonomous motivation in leisure time | 4 | 5.84 | 1.24 | .913 |
| Controlled motivation in PE | 4 | 2.99 | 1.16 | .548 |
| Controlled motivation in leisure time | 4 | 3.17 | 1.42 | .796 |
| Amotivation in PE | 2 | 2.47 | 1.58 | .750 |
| Amotivation in leisure time | 2 | 2.03 | 1.37 | .751 |
| Attitudes | 3 | 6.05 | 1.02 | .803 |
| Intentions | 2 | 5.60 | 1.32 | .898 |
| Perceived behavioral control | 2 | 6.02 | 1.02 | .758 |
| Subjective norms | 2 | 4.72 | 1.44 | .808 |
| Automaticity | 4 | 5.08 | 1.39 | .852 |
| Self-discipline | 10 | 2.91 | 0.53 | .861 |
| Grit | 12 | 2.79 | 0.43 | .751 |
| Teachers’ perceived provision of autonomy support | 8 | 5.37 | 0.55 | .718 |
| Teachers’ perceived provision of control | 3 | 3.74 | 0.96 | .530 |

*Note*. PE = Physical education. Physical activity is presented as MET-minutes. Physical activity MET-minutes were calculated by multiplying days of doing each type of physical activity × minutes spent doing each type of physical activity × MET value. MET values were set at 3.3 for walking, 4.0 for MPA, and 8.0 for VPA [67].

**Table S5**

*Pearson’s Correlations Between Key Variables at Baseline*

|  | 2 | 3 | 4 | 5 | 6 | 7 | 8 | 9 | 10 | 11 | 12 | 13 | 14 | 15 | 16 | 17 | 18 | 19 | 20 | 21 | 22 | 23 |
| --- | --- | --- | --- | --- | --- | --- | --- | --- | --- | --- | --- | --- | --- | --- | --- | --- | --- | --- | --- | --- | --- | --- |
| 1. Vigorous PA | **.375^**^** | **.196^**^** | **.714^**^** | .109^*^ | **.140^**^** | **.151^**^** | **.215^**^** | **.249^**^** | **.498^**^** | -.090 | .037 | -.006 | -.105^*^ | **.280^**^** | **.456^**^** | **.347^**^** | **.121^*^** | **.387^**^** | **.245^**^** | **.275^**^** | -.057 | .104^*^ |
| 2. Moderate PA |  | **.220^**^** | **.621^**^** | .091 | **.122^*^** | **.151^**^** | **.122^*^** | **.217^**^** | **.320^**^** | -.063 | -.015 | -.048 | -.025 | **.212^**^** | **.318^**^** | **.269^**^** | **.164^**^** | **.268^**^** | **.224^**^** | **.272^**^** | -.075 | **.112^*^** |
| 3. Walking |  |  | **.481^**^** | **.113^*^** | .020 | .082 | .090 | **.144^**^** | **.269^**^** | -.093 | .002 | -.050 | -.045 | **.169^**^** | **.215^**^** | **.136^**^** | .019 | **.240^**^** | **.156^**^** | .068 | **-.126^*^** | -.065 |
| 4. Total PA |  |  |  | **.144^*^** | **.128^*^** | **.146^**^** | **.196^**^** | **.264^**^** | **.523^**^** | **-.129^*^** | -.011 | .025 | -.063 | **.311^**^** | **.499^**^** | **.340^**^** | **.146^**^** | **.478^**^** | **.295^**^** | **.324^**^** | -.081 | .047 |
| 5. PAS |  |  |  |  | -.109^*^ | **.376^**^** | **.376^**^** | **.443^**^** | **.352^**^** | -.058 | -.075 | **-.161^**^** | **-.127^*^** | **.324^**^** | **.300^**^** | **.279^**^** | .090 | **.233^**^** | **.313^**^** | **.284^**^** | **-.172^**^** | .095 |
| 6. PC |  |  |  |  |  | .045 | .032 | .052 | **.141^**^** | .102^*^ | .096 | **.116^*^** | .059 | .055 | .093 | .037 | **.114^*^** | **.131^*^** | .066 | -.030 | .077 | .063 |
| 7. PASPAR |  |  |  |  |  |  | **.389^**^** | **.338^**^** | **.421^**^** | -.104^*^ | -.074 | **-.166^**^** | **-.193^**^** | **.377^**^** | **.399^**^** | **.381^**^** | **.195^**^** | **.251^**^** | **.378^**^** | **.423^**^** | -.073 | .083 |
| 8. PASPEER |  |  |  |  |  |  |  | **.332^**^** | **.420^**^** | -.063 | -.045 | -.058 | -.099 | **.334^**^** | **.376^**^** | **.230^**^** | .108^*^ | **.312^**^** | **.240^**^** | **.267^**^** | -.080 | -.023 |
| 9. AUTMOT (PE) |  |  |  |  |  |  |  |  | **.616^**^** | -.093 | .010 | **-.238^**^** | **-.182^**^** | **.396^**^** | **.489^**^** | **.247^**^** | **.166^**^** | **.468^**^** | **.460^**^** | **.338^**^** | **-.155^**^** | -.110^*^ |
| 10. AUTMOT (LT) |  |  |  |  |  |  |  |  |  | -.072 | .025 | **-.157^**^** | **-.246^**^** | **.580^**^** | **.739^**^** | **.397^**^** | **.181^**^** | **.643^**^** | **.464^**^** | **.516^**^** | **-.129^*^** | -.045 |
| 11. CONMOT (PE) |  |  |  |  |  |  |  |  |  |  | **.349^**^** | **.241^**^** | **.195^**^** | -.003 | **-.120^*^** | -.070 | .078 | **-.122^*^** | **-.209^**^** | **-.167^**^** | .009 | .028 |
| 12. CONMOT (LT) |  |  |  |  |  |  |  |  |  |  |  | **.195^**^** | **.298^**^** | -.027 | -.015 | -.105^*^ | **.358^**^** | **-.175^**^** | **-.134^*^** | **-.176^**^** | .070 | .020 |
| 13. AMOT (PE) |  |  |  |  |  |  |  |  |  |  |  |  | **.566^**^** | **-.177^**^** | **-.151^**^** | -.094 | .003 | **-.158^**^** | **-.275^**^** | **-.257^**^** | -.063 | .005 |
| 14. AMOT (LT) |  |  |  |  |  |  |  |  |  |  |  |  |  | **-.279^**^** | **-.229^**^** | **-.217^**^** | .055 | **-.240^**^** | **-.239^**^** | **-.248^**^** | -.083 | .065 |
| 15. Attitudes |  |  |  |  |  |  |  |  |  |  |  |  |  |  | **.597^**^** | **.445^**^** | **.183^**^** | **.401^**^** | **.328^**^** | **.359^**^** | -.037 | .006 |
| 16. Intentions |  |  |  |  |  |  |  |  |  |  |  |  |  |  |  | **.590^**^** | **.285^**^** | **.592^**^** | **.465^**^** | **.474^**^** | -.037 | -.044 |
| 17. PBC |  |  |  |  |  |  |  |  |  |  |  |  |  |  |  |  | **.174^**^** | **.387^**^** | **.331^**^** | **.363^**^** | -.024 | .059 |
| 18. Sub. norms |  |  |  |  |  |  |  |  |  |  |  |  |  |  |  |  |  | **.179^**^** | **.173^**^** | .108^*^ | -.027 | .000 |
| 19. Automaticity |  |  |  |  |  |  |  |  |  |  |  |  |  |  |  |  |  |  | **.456^**^** | **.466^**^** | **-.133^*^** | .043 |
| 20. Self-discipline |  |  |  |  |  |  |  |  |  |  |  |  |  |  |  |  |  |  |  | **.674^**^** | **-.120^*^** | .064 |
| 21. Grit |  |  |  |  |  |  |  |  |  |  |  |  |  |  |  |  |  |  |  |  | **-.147^**^** | .080 |
| 22. TPRAS |  |  |  |  |  |  |  |  |  |  |  |  |  |  |  |  |  |  |  |  |  | **-.203^**^** |
| 23. TPRC |  |  |  |  |  |  |  |  |  |  |  |  |  |  |  |  |  |  |  |  |  |  |

*Note*. AMOT (LT) = Amotivation (in leisure time); AMOT (PE) = Amotivation (in physical education); AUTMOT (LT) = Autonomous motivation (in leisure time); AUTMOT (PE) = Autonomous motivation (in physical education); CONMOT (LT) = Controlled motivation (in leisure time); CONMOT (PE) = Controlled motivation (in physical education); PA = Physical activity; PAS = Perceived autonomy support; PASPAR = Perceived autonomy support from parents; PASPEER = Perceived autonomy support from peers; PBC = Perceived behavioral control; PC = Perceived control; TPRAS = Teachers’ perceived provision of autonomy support; TPRC = Teachers’ perceived provision of control. Values that remained significant when adjusted for family wise error rate using the false detection method are highlighted in bold (FDR: α = .05, *q* = .05, *m* = 253).

^*^*p* <.05. ^**^*p* <.01.

# Appendix G

**Table S6**

*Descriptive Statistics for Trans-Contextual Model Constructs over Time*

|  | Measurement Occasion | Intervention Group  (*N* = 174) | | Waitlist Control Group  (*N* = 196) | |
| --- | --- | --- | --- | --- | --- |
| *Variable* |  | *M* | *SD* | *M* | *SD* |
| Perceived autonomy support | Baseline | 5.73 | 0.82 | 5.64 | 0.95 |
|  | Post-intervention | 5.64 | 0.90 | 5.56 | 1.06 |
|  | One month | 5.53 | 0.93 | 5.58 | 1.05 |
| Autonomous motivation (PE) | Baseline | 5.40 | 1.48 | 5.74 | 1.21 |
|  | Post-intervention | 5.38 | 1.31 | 5.63 | 1.24 |
|  | One month | 5.35 | 1.41 | 5.68 | 1.21 |
| Autonomous motivation (LT) | Baseline | 5.86 | 1.25 | 5.83 | 1.24 |
|  | Post-intervention | 5.83 | 1.19 | 5.87 | 1.16 |
|  | One month | 5.82 | 1.20 | 5.87 | 1.20 |
| Attitudes | Baseline | 6.11 | 1.06 | 5.99 | 0.98 |
|  | Post-intervention | 6.00 | 1.10 | 5.92 | 1.00 |
|  | One month | 5.80 | 1.14 | 6.12 | 0.93 |
| Subjective norms | Baseline | 4.76 | 1.36 | 4.68 | 1.51 |
|  | Post-intervention | 4.93 | 1.26 | 4.66 | 1.39 |
|  | One month | 4.64 | 1.41 | 4.66 | 1.49 |
| Perceived behavioral control | Baseline | 6.06 | 1.02 | 5.98 | 1.02 |
|  | Post-intervention | 6.05 | 0.98 | 5.90 | 0.91 |
|  | One month | 5.73 | 1.07 | 5.97 | 0.94 |
| Intentions | Baseline | 5.59 | 1.42 | 5.60 | 1.23 |
|  | Post-intervention | 5.79 | 1.19 | 5.64 | 1.18 |
|  | One month | 5.59 | 1.36 | 5.85 | 1.10 |
| Physical activity^a^ | Baseline | 6357.89 | 4288.37 | 6827.14 | 4130.26 |
|  | Post-intervention | 5414.79 | 3829.93 | 5728.00 | 3844.72 |
|  | One month | 5266.24 | 3476.71 | 5926.59 | 4268.20 |

*Note*. LT = Leisure time; PE = Physical education.

^a^Post-intervention sample sizes: intervention group, *N* = 137 and waitlist control group, *N* = 172; One month follow-up: intervention group, *N* = 129 and waitlist control group, *N* = 158.
